# Vaginal Lymphoma: A Possible Cause of Genital Hemorrhage

**DOI:** 10.4274/tjh.2015.0112

**Published:** 2016-08-19

**Authors:** Erdoğan Nohuz, Sharif Kullab, Albane Ledoux-Pilon, Cécile Moluçon-Chabrot, Maël Albaut, Luisa De Simone, Xavier Durando

**Affiliations:** 1 General Hospital of Thiers, Clinic of Obstetrics and Gynecology, Thiers, France; 2 Centre Jean Perrin, Clinic of Medical Oncology, Clermont-Ferrand, France; 3 Estaing University Hospital, Department of Pathology, Clermont-Ferrand, France; 4 Estaing University Hospital, Department of Hematology, Clermont-Ferrand, France

**Keywords:** non-Hodgkin’s lymphoma, Vaginal B-cell lymphoma, Postmenopausal bleeding, Vaginal discharge

A 59-year-old patient complaining of vaginal bleeding and puruloid discharge was admitted to our gynecology department. Speculum examination showed a vaginal fungating necrotic ulcerated mass. There was no palpable lymphadenopathy or hepato-splenomegaly on physical examination. Transvaginal ultrasound and abdominopelvic computed tomography demonstrated a bulky vaginal mass approximately 5x4x3 cm in diameter involving the bladder and the rectovaginal septum. With the patient’s approval, a punch biopsy was performed and failed to establish the diagnosis (small and necrotic samples that were not representative of the lesion).

Histopathological diagnosis was obtained after a second biopsy performed under general anesthesia. Immunohistochemistry showed that tumor cells were positive for CD20, CD30, MUM1, and bcl-6 and were negative for bcl-2, EMA, CD10, and CD30 (Figure 1). The patient was diagnosed with primary vaginal diffuse large-B-cell non-Hodgkin lymphoma (NHL) and underwent 8 courses of rituximab, cyclophosphamide, doxorubicin, vincristine, prednisone immunochemotherapy. Complete remission was achieved without any relapse at 18 months’ follow-up.

Primary vaginal NHL represents less than 1% of genital neoplasms [1,2,3]. Early and accurate diagnosis significantly influences the prognosis [4,5]. It should be considered in differential diagnosis of patients with vaginal bleeding. A deep biopsy may be required.

## Ethics

Informed Consent: It was taken.

## Figures and Tables

**Figure 1:**
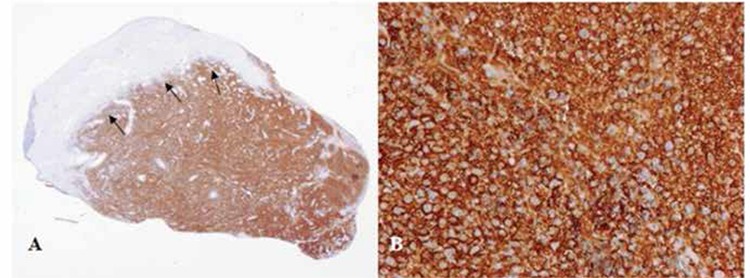
A) Grenz zone (arrows): CD20-positive immunoreactivity in neoplastic cells (25^x^). B: Immunohistochemical analysis of paraffin-embedded sections of the mass lesion showing tumor cells expressing the CD20 molecule (400^x^).
